# Efficacy and Safety of Oral Herbal Drugs Used as Adjunctive Therapy for Melasma: A Systematic Review and Meta-Analysis of Randomised Controlled Trials

**DOI:** 10.1155/2021/9628319

**Published:** 2021-12-06

**Authors:** Qingti Tang, Hongjie Yang, Xiarong Liu, Yu Zou, Xintong Lv, Kai Chen

**Affiliations:** ^1^Department of Dermatology, The Affiliated Hospital of Chengdu University, Chengdu, China; ^2^Department of Radiology, The Sixth People's Hospital of Chengdu, Chengdu, China; ^3^Department of Pharmacy, Taizhou People's Hospital Affiliated to Nanjing University of Traditional Chinese Medicine, Taizhou, China

## Abstract

**Background:**

Melasma is an acquired disorder of facial pigmentation. Its etiology is multifactorial; thus, the management is usually challenging. As a complementary therapy, herbal drugs are often used in the management of melasma. This work was aimed to investigate the efficacy and safety of herbal drugs on melasma in female patients.

**Methods:**

This study followed Preferred Reporting Items for Systematic Reviews and Meta-Analyses (PRISMA) guidelines. A comprehensive search was conducted, and all randomised controlled trials (RCTs) on the use of oral herbal drugs as complementary therapy for melasma in female patients were included. A meta-analysis was conducted according to the guidelines of the Cochrane Collaboration using Review Manager 5.4.

**Results:**

Ten eligible trials, with 1015 female melasma patients, were included. All of the included RCTs had some concerns for risk of bias for different reasons, especially for that most of included trials were unblinded. Pooled data suggested phytotherapy plus routine therapy had significantly better efficacy on melasma than routine therapy, in terms of response rate (OR: 4.49, 95% CI: 3.25 to 6.20, *p* < 0.00001), reduction of skin lesion score (SMD: −0.56, 95% CI: −0.79 to −0.33, *p* < 0.00001), and improvement of serum E2 levels (SMD: −1.58, 95% CI: −2.62 to −0.55, *p* 0.003). In addition, there was no significant difference in the incidence of AEs between phytotherapy plus routine therapy and routine therapy (OR: 0.92, 95% CI: 0.53 to 1.58; p 0.76). Overall, herbal drugs used as an adjunct to routine therapy significantly enhanced the efficacy for the treatment of melasma but with a comparable safety profile.

**Conclusion:**

These findings have implications for recommending herbal drugs as a viable complementary treatment option for melasma.

## 1. Introduction

Melasma is an acquired disorder of facial pigmentation characterized by irregular tan or brown macules on the forehead, cheeks, and upper lip. Epidemiologic studies have estimated the prevalence of melasma in different populations, and it varies according to skin types, ethnic composition, and levels of UV exposure [1–4]. It is estimated that the prevalence of melasma in the general population is 1%, while in the high-risk population it is 9–50% [1–4]. The melasma-prone population includes East Asians (Chinese, Korean, and Japanese), Indians, Pakistanis, and Middle Easterners [5]. Although the exact pathogenesis of melasma has not yet been elucidated, hormone secretion, genetic factors, and chronic ultraviolet (UV) exposure are reported to play important roles in its occurrence [6–8]. Other studies also suggest that skin inflammation is involved in the pathogenesis of melasma [5, 9]. Melanin synthesis is a tyrosinase-dependent process consisting of tyrosine hydrolysis to L-DOPA, DOPA oxidation to quinine, and quinine oxidation to melanin [6–8]. Tyrosinase-related proteins (TRP) include tyrosinase, TRP-1 and TRP-2. Microphthalmia-associated transcription factor (MITF) plays a fundamental role in the transcriptional regulation of these genes. A thorough understanding of the pathogenesis of melasma is crucial to the appropriate management of melasma.

Since the etiology of melasma is multifactorial, the management of this condition is usually challenging. Currently, the gold standard treatment for melasma is topical hydroquinone cream and broad-spectrum sunscreens. Hydroquinone is a tyrosinase inhibitor for blocking the conversion of DOPA to melanin. However, hydroquinone has been associated with the development of exogenous ochronosis and mutagenicity [10, 11]. Off-label tranexamic acid has emerged as a potential treatment for melasma since it was suggested to inhibit melanin synthesis by blocking the interaction between melanocytes and keratinocytes [12, 13]. Other commonly used agents include vitamin C and glutathione. Although various treatment modalities have been used, with inconsistent results, the efficacy is often insufficient.

Herbal medicines have been used empirically in topical therapy since ancient times. Both patients and physicians are increasingly welcoming the use of herbal medicines as an adjunct to routine therapy in view of their presumed high tolerance and efficacy. The herbal prescription for oral administration has been reported to function to improve endocrine secretion, tone the kidneys, relieve the depressed liver, and regulate the circulation of blood, which has been increasingly used as an adjunctive treatment of various skin conditions [14, 15]. Recently, randomised controlled trials (RCTs) have been conducted on the effects of using herbal drugs as an adjunctive therapy on the improvement of melasma. However, there is a lack of sufficiently pooled evidence on their efficacy and safety. In this study, we conducted a systematic review and meta-analysis of RCTs to investigate the efficacy and safety of applying herbal drugs as adjunctive therapy in patients with melasma.

## 2. Materials and Methods

This study was conducted and reported according to Preferred Reporting Items for Systematic Reviews and Meta-Analyses (PRISMA) guidelines [16]. The study protocol was prospectively registered on PROSPERO (CRD42021283700).

### 2.1. Search Strategy

Seven databases (PubMed, Embase, the Cochrane Library, Web of Science, Scopus, CNKI, and WANFANG data) were systematically searched from the inception of the databases until October 2021. This search was conducted by two independent reviewers (X. R. Liu and Y. Zou). Studies on melasma were identified with the terms *melanosis* (as a medical subject heading (MeSH) and a free text term), and *melasma, chloasma, melanose$, melanism*, or *freckle* (as free text terms). These were combined using the set operator and with studies identified with the terms: *herbal medicine, medicinal plants, herbal drugs, plant extracts, or phytotherapy* (as MeSH terms and free text terms), and the following terms: *herb$, plant^∗^, botanical, natural product, weed∗, algae, fungi, or fungus* (as free text terms). A manual citation check of included articles was also performed to find any additional studies.

### 2.2. Study Selection

Inclusion criteria were as follows: RCTs describing the efficacy and safety of oral herbal medicines used as an adjunct in the treatment of melasma in females. Participants included in trials should be healthy adults with melasma diagnosed by dermatological examination. We excluded studies that provided no gender information of participants and studies that included participants with pregnancy or breastfeeding. According to the defined criteria, 2 authors (Q. T. Tang and H. J. Yang) independently selected records based on the title and abstract and then performed an eligibility assessment based on the full text. Any discrepancies between investigators were resolved by discussion to reach consensus.

### 2.3. Study Outcomes

The primary outcome was physician-assessed improvement in melasma reported as a response rate. The secondary outcomes included melasma improvement evaluated through the changes of Melasma Area and Severity Index (MASI) score, skin lesion score, and serum levels of estradiol (E2), as well as AEs occurring as a result of therapy.

### 2.4. Data Extraction

For each included trial, 2 authors (Q. T. Tang and H. J. Yang) independently extracted information on the first author, publication year, country, study design, characteristics of patients, description of intervention, outcomes, and duration of follow-up. These data were extracted using a standard data extraction form, with any disagreement resolved by discussion to reach a consensus. For missing data, the first author of the report was contacted when possible.

### 2.5. Risk of Bias Assessment

The risk of bias and methodological quality of included RCTs were assessed using the Cochrane Collaboration's “risk of bias” tool as outlined in the Cochrane Handbook for Systematic Reviews of Interventions [17]. Two investigators (Q. T. Tang and H. J. Yang) independently performed the assessment of each study, with any disagreement resolved by discussion to reach a consensus.

### 2.6. Data Synthesis and Analysis

Data collected from trials were preprocessed within Microsoft Excel. Meta-analyses were conducted using Review Manager (RevMan) Version 5.4 (Cochrane Collaboration). The outcomes including the response rate and AEs were dichotomous data while the outcomes including the changes of MASI score, skin lesion score, and E2 levels were continuous data. Pooled dichotomous data were expressed as odds ratio (OR) with 95% confidence interval (CI). Pooled continuous data were expressed as standardized mean difference (SMD) with 95% CI. Heterogeneity was assessed by virtually examining the forest plot to detect nonoverlapping CIs using the *χ*^2^ test of heterogeneity (with *p* < 0.1 indicating statistical significance) and the *I*^2^ statistic of inconsistency (with <30%, 30%–60%, and >60%, respectively, representing low, moderate, or high heterogeneity). A *p* value of <0.05 was considered significant for the test of overall effect. Funnel plots were used to assess potential small study effects when at least 10 trials were available within a comparison. Meanwhile, publication bias was also assessed by the Egger test, with *p* < 0.05 indicating statistical significance.

## 3. Results

### 3.1. Literature Search

**Figure 1:**
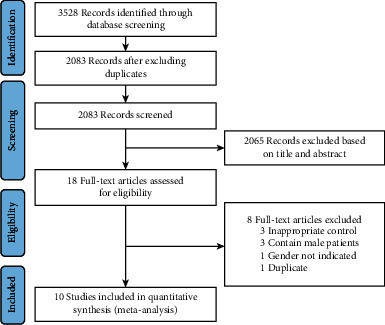
Study flow diagram.

The literature search yielded 3528 records in which 2083 records were retained after removing duplicates. After the first screening based on title and abstract, 2065 records were excluded. The full text of 18 studies was reviewed for inclusion. After excluding studies that did not meet inclusion criteria, 10 studies were deemed appropriate and were included for meta-analysis [18–27]. The process for study selection is shown in Figure 1.

### 3.2. Study Characteristics

The characteristics of the included studies are summarized in Table 1. Ten trials enrolled 1015 female participants. All the study participants were adults with ages ranging from 19 to 55 years. All trials were completed in China during 2013–2019. Of 10 trials, 9 trials reported the duration of melasma in patients [18, 19, 21–27], while 1 trial did not [20]. Across the included trials, the routine regimens for melasma treatment varied. Topical therapies alone were applied in 2 trials [18, 20], oral administration alone was used in 4 trials [21, 23, 27], and topical therapies combined oral administration were applied in 4 trials [19, 24–26]. Among routine regimens, the predominant topical drug was hydroquinone cream, and the predominant oral drug was glutathione tablet. As an adjunct to routine therapy, the regimens of oral herbal medicines differed among these studies. Of 10 trials, 3 trials used Danggui Shaoyao preparation as an adjuvant [18, 22, 27], 3 trials used Honghua Xiaoyao tablet [19, 21, 26], 1 trial used Bazhen capsule [20], 1 trial used Jingtian Quban capsule [23], 1 trial used Tiaogan Jianpi Quban powder [24], and 1 trial used Tiaochong Xiaoban decoction [25]. Among these drugs, Honghua Xiaoyao tablet was administered three times daily, while other drugs were administered twice-daily. The duration of intervention was 12 weeks for all trials. A total of 5 trials reported the incidence of AEs [19, 21, 26, 27]. Across studies, the outcome measures included physician assessment, melasma area and color score, serum levels of sex hormones and biochemical indexes, MASI score, and dermatology life quality index (DLQI). A descriptive summary of study outcomes and efficacy is shown in Table 2.

### 3.3. Risk of Bias Assessment

**Figure 2:**
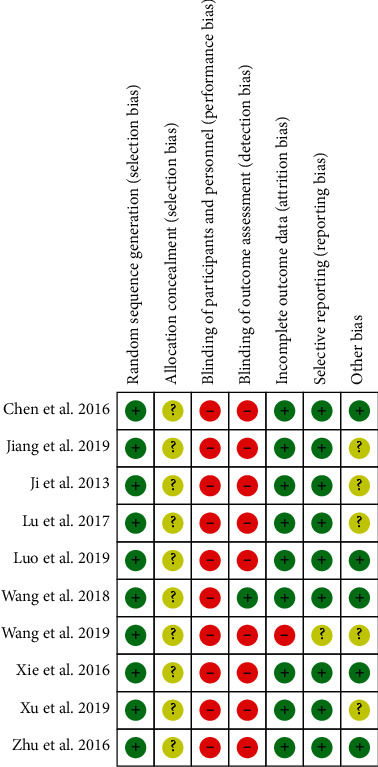
Risk of bias assessment.

Figure 2 shows the detailed assessment of the risk of bias. All of the included RCTs had some concerns for risk of bias for different reasons. All studies claimed to be randomised and presented similar baseline characteristics between groups, but failed to provide adequate information on allocation concealment. Of the 10 RCTs included, only 1 study claimed to be single-blind [24] while the others were unblinded. Furthermore, all studies did not provide adequate information to determine whether the outcome assessors were blinded or not. One study provided incomplete outcome data due to the absence of serum biochemical data at baseline [23]. Of 10 RCTs included, 5 studies used herbal drugs produced from commercial industries, but did not provide adequate information on funding [19, 21, 23, 26]. In summary, the quality of the included studies varied but was generally poor.

### 3.4. Meta-Analysis of Efficacy Outcome

**Figure 3:**
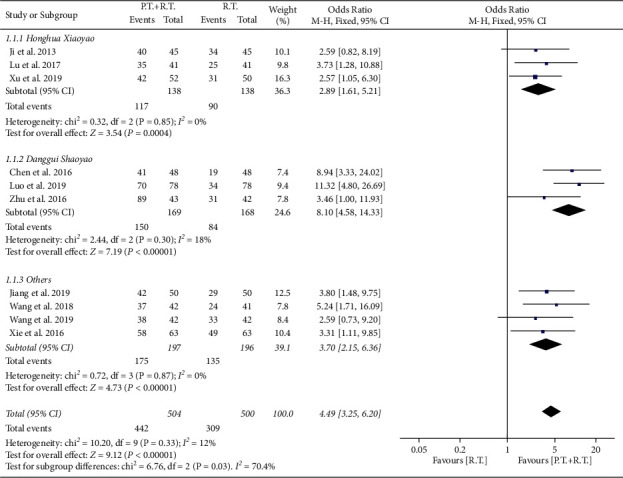
Forest plot of response rate in the overall analysis. Subgroup analysis was stratified according to the types of adjunct drugs. PT + RT = phytotherapy plus routine therapy; RT = routine therapy.

A total of 10 studies reported the response rates were included in the meta-analysis for the primary outcome. In total, there were 1004 patients, 504 of whom received routine therapy with phytotherapy as an adjunct, and another 500 received routine therapy alone. As shown in Figure 3, pooling analysis suggested a significant difference in response rates between phytotherapy plus routine therapy and routine therapy, showing a pooled OR of 4.49 (95% CI 3.25 to 6.20, *p* < 0.00001). Subgroup analysis was also conducted according to the types of adjunct drugs. The effect size of Honghua Xiaoyao (OR: 2.89, 95% CI: 1.61 to 5.21, and p 0.004) was smaller than overall effect size, while the effect size of Danggui Shaoyao (OR 8.10, 95% CI 4.58 to 14.33, *p* < 0.00001) was larger than overall effect size. The heterogeneity across studies was found to be low (*χ*^2^ = 10.20, df = 9, *p*=0.33, *I*^2^ = 12%). The tolerable symmetry of funnel plot (Supplementary Figure S1) and the result of Egger test (*p*=0.003) suggested low risk of publication bias or other small study effects.

**Figure 4:**
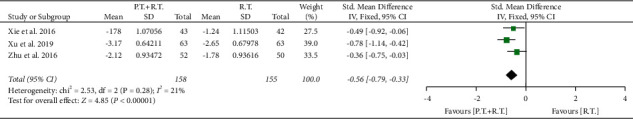
Forest plot of change in skin lesion score in the overall analysis. PT + RT = phytotherapy plus routine therapy; RT = routine therapy.

**Figure 5:**
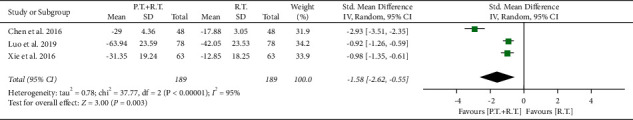
Forest plot of change in serum estradiol (E2) levels in overall analysis. PT + RT = phytotherapy plus routine therapy; RT = routine therapy.

Three studies containing 158 participants in the phytotherapy plus routine therapy group and 155 in the routine therapy group, reported the efficacy through the changes of skin lesion score [25–27]. Pooled analysis suggested that combination therapy had a greater effect on the reduction of skin lesion score than routine therapy (SMD −0.56, 95% CI −0.79 to −0.33, *p* < 0.00001; Figure 4). Likewise, the meta-analysis from 2 studies [22, 24] showed that MASI reduction was greater in the phytotherapy plus routine therapy group compared with the routine therapy group (SMD: −2.20, 95% CI: −3.92 to −0.48, and p 0.01; Supplementary Figure S2). In 3 studies [18, 22, 25], the changes of serum E2 levels were reported between baseline and end-point. Phytotherapy plus routine therapy caused a greater reduction of E2 levels versus routine therapy alone (SMD: −1.58, 95% CI: −2.62 to −0.55, and *p* 0.003; Figure 5).

### 3.5. Meta-Analysis of Safety Outcome

**Figure 6:**
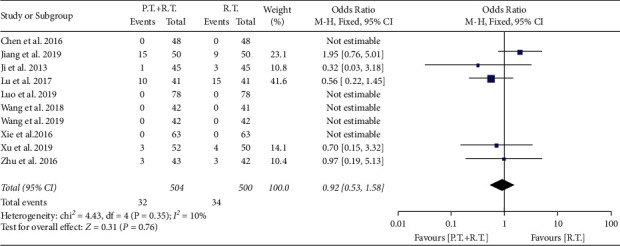
Forest plot of adverse events (AEs) in the overall analysis. PT + RT = phytotherapy plus routine therapy; RT = routine therapy.

In 10 trials, AEs were identified in 5 studies (Table 1), but no serious AEs were reported. The majority of AEs were gastrointestinal reactions which were reported in 5 studies [19, 21, 26, 27], and others were pruritus [20], erythema [19, 20], pricking [27], peeling [27], menoxenia [21, 26] and burning [19]. There were 32 (6.3%) of 504 patients receiving combination therapy who experienced AEs, compared with 34 (6.8%) of 500 patients receiving routine therapy. Pooled data showed that there was no significant difference in the incidence of AEs between patients receiving phytotherapy plus routine therapy and routine therapy (OR: 0.92, 95% CI: 0.53 to 1.58; p 0.76; Figure 6). Overall, phytotherapy plus routine therapy was well tolerated across studies. The symmetry of the funnel plot (Supplementary Figure S3) and the Egger test (p 0.7432) suggested the small potentiality of publication bias.

## 4. Discussion

Although various therapy options have been offered, no regimen guarantees satisfactory results. Currently, management of melasma remains challenging, and the exploitation of safe and effective therapies is imperative. Herbal medicines have been used empirically as therapeutic agents since ancient times. Treating aesthetically displeasing skin disorders using herbal drugs as an adjunct is gaining interest due to their high tolerance and efficacy as well as their perception of safety [28–30]. Only recently, though, the clinical efficacy of some of these herbal drugs has been substantiated through clinical studies. In this regard, our aim was to examine the efficacy and safety of various herbal drugs used as adjuncts to routine agents in the treatment of melasma. We found that combination therapy consisting of phytotherapy and routine therapy significantly increased the response rate when compared with routine therapy alone. In addition, the meta-analysis from 3 studies [25–27] suggested that the effect sizes of combination therapy for skin lesion scores were larger than routine therapy alone. Besides those subjective outcome measures, objective outcome measures, such as serum levels of sex hormones, were also used in several studies [18, 19, 22, 24, 25, 27]. From the pooled results of 3 studies [18, 22, 25], we found that there was a significant benefit associated with the use of herbal drugs as an adjunct to routine therapy in serum E2 levels when compared with routine therapy alone. These results were consistent with the findings from pooled subjective outcomes. Regarding the safety of herbal drugs, our systematic review demonstrated that herbal drugs were well tolerated, with only a small proportion of patients receiving herbal drugs experiencing AEs. In addition, our meta-analysis also suggested that rates of AEs for routine therapy with herbal drugs as an adjunct were comparable to those for routine therapy alone.

Across included studies, there was no sufficient data for assessing the efficacy of phytotherapy plus routine therapy at different end-points. However, in one trial [20], we noted an increase in response rate at the 12th week when compared with the 8th week, suggesting the duration of intervention is important for combination therapy to achieve better therapeutic effect. As previously reported, melasma is easy to recrudesce making efficacy maintenance challenging [31]. One of the included trials reported the recurrence rate at 3 months' follow-up, and the recurrence rate was significantly lower in the combination therapy group than in the routine therapy group [20]. Moreover, another trial demonstrated that phytotherapy plus routine therapy caused a significant decrease in MASI and a significant increase in DLQI at the 12th week, with no rebound within 3 months of combination therapy cessation [24]. By contrast, within 3 months of routine therapy cessation, the reduction of MASI and increase of DLQI were receded [24]. These results suggested that phytotherapy plus routine therapy could have an advantage over routine therapy in efficacy maintenance.

Of the included trials, the agents used in routine therapy mainly included hydroquinone, tranexamic acid, glutathione, and vitamin C. The action mechanisms underlying the efficacy of routine therapy vary with the contained agents. Hydroquinone can bind to the active site of tyrosinase and inhibit the conversion of 3,4-dihydroxyphenylalanine (DOPA) to melanin [31]. Tranexamic acid decreases the generation of arachidonic acid, which leads to a reduction in melanocyte-stimulating hormone and a decrease in pigmentary production [32]. Vitamin C possesses antioxidant activity to scavenge free radicals, while glutathione is thought to act as an antioxidant that decreases inflammation [33]. Differ to the abovementioned agents, the efficacy of herbal drugs were suggested to be derived from the synergetic and holistic functions of active ingredients rather than the effect on a single target. The herbal drugs were reported to improve the functions of the liver and spleen, dredge the channels and collaterals, and promote blood circulation to remove blood stasis so as to extinct the facial pigmented spots [18, 19, 21, 22, 26]. In addition, herbal drugs could improve internal secretion to regulate the levels of progesterone, E2, and testosterone [18, 22, 25]. Some studies suggested that active ingredients from herbal drugs exerted antioxidant and anti-inflammatory effects which could contribute to the inhibition of melanocyte proliferation [18, 19, 21, 22]. In summary, the function and underlying mechanism of herbal drugs were believed to be complementary to routine therapy, thus resulting in a synergistic effect on the improvement of melasma.

There were some limitations deserving for discussion in this work. First, the quality of included trials was generally poor. Secondly, the included trials and enrolled participants for each meta-analysis were relatively small. Thirdly, the duration of intervention was short, within 12 weeks, there was a lack of data on long-term efficacy. Despite the discussed limitations, this work provides a snapshot of the best level of evidence currently available on the use of oral herbal drugs as an adjunct in the treatment of melasma.

In conclusion, our systematic review and meta-analysis investigated the efficacy and safety of oral herbal drugs used as adjunctive therapy for melasma. Herbal drugs are increasingly popular supplements to standard therapy agents. Evidence-based knowledge of the efficacy and safety will be helpful for dermatologists who may prescribe these herbal drugs. Our results demonstrated that herbal drugs used as an adjunct to routine therapy significantly enhanced the response rate in the treatment of melasma (OR: 4.49, 95% CI: 3.25 to 6.20, *p* < 0.00001). In addition, such combination therapy was also well tolerated with a comparable safety profile to routine therapy alone (OR: 0.92, 95% CI: 0.53 to 1.58; *p* 0.76). These findings might have implications for recommending herbal drugs as a viable complementary treatment option for melasma. However, the poor design of these included studies has hampered the reliability of these findings. Moreover, none of the included studies provided long-term effects of the interventions and incorporated participant assessment data. Therefore, more rigorous clinical studies with longer follow-up durations need to be conducted to strengthen the reliability of these findings.

## Figures and Tables

**Table 1:** Characteristics of included studies.

Interventions								
Study	Study design	Sample size	Age (years)	Duration (years)	Experiment group (E)	Control group (C)	Adverse events	Dropout (reason)
Chen et al. 2016	Randomized, controlled trial	96 (48E, 48C)	19–43 E: 32.6 ± 6.4; C: 31.8 ± 6.9	E: 2.6 ± 1.4; C: 2.8 ± 1.5	Danggui Shaoyao powder, twice-daily + ultrasonic therapy with L-vitamin C, once-weekly for 12 weeks	Ultrasonic therapy with L-vitamin C, once-weekly for 12 weeks	NR	0

Ji et al. 2013	Randomized, controlled trial	100 (50E, 50C)	28–50 40.33 ± 6.35	2–22 13.2 ± 2.1	Honghua Xiaoyao tablet, three times daily + vitamin C, three times daily, and hydroquinone cream, twice-daily for 12 weeks	Vitamin C, three times daily, and hydroquinone cream, twice-daily for 12 weeks	E: 9 × gastrointestinal reaction, 6 × burning and erythema; C: 9 × burning and erythema	0

Jiang et al. 2019	Randomized, controlled trial	90 (45E, 45C)	29–51 E: 45.52 ± 4.39; C: 45.63 ± 4.42	NR	Ba Zhen capsule, twice-daily + hydroquinone cream, twice-daily for 12 weeks	Topical hydroquinone cream, twice-daily for 12 weeks	E: 1 × gastrointestinal reaction; C: 1 × pruritus and 2 × erythema	0

Lu et al. 2017	Randomized, controlled trial	82 (41E, 41C)	29–55 E: 42.4 ± 6.5; C: 41.1 ± 7.7	E: 8.9 ± 2.8; C: 10.0 ± 2.3	Honghua Xiaoyao granule, three times daily + tranexamic acid tablet, twice-daily for 12 weeks	Tranexamic acid tablet, twice-daily for 12 weeks	E: 7 × gastrointestinal reaction and 3 × menoxenia; C: 8 × gastrointestinal reaction and 7 × menoxenia	0

Luo et al. 2019	Randomized, controlled trial	156 (78E, 78C)	21–45 E: 34.6 ± 2.5; C: 33.7 ± 2.4	E: 4.2 ± 1.9; C: 5.3 ± 1.7	Danggui Shaoyao powder, twice-daily + glutathione, three times daily, and vitamin C, three times daily, and vitamin E, once-daily for 12 weeks	Glutathione tablets three times daily, and vitamin C tablets, three times daily, and vitamin E capsules, once-daily for 12 weeks	0	0

Wang et al. 2018	Randomized evaluator-blinded clinical trial	94 (47E, 47C)	24–49 E: 36.36 ± 7.43; C: 35.61 ± 6.69	E: 3.46 ± 2.62; C: 3.52 ± 2.25	Tiaogan Jianpi Quban powder, twice-daily + vitamin C tablet, three times daily, and vitamin E cream, once-daily for 12 weeks	Vitamin C tablets, three times daily and vitamin E cream, once-daily for 12 weeks	0	E: 5 (unknown reasons); C: 6 (unknown reasons)

Xie et al. 2016	Randomized, controlled trial	126 (63E, 63C)	30–48 E: 37.3 ± 9.1; C: 38.1 ± 8.6	E: 1.6 ± 0.9; C: 1.7 ± 1.0	Tiaochong Xiaoban decoction, twice-daily + glutathione, and vitamin C and vitamin E tablets, three times daily, and hydroquinone cream, twice-daily for 12 weeks	Glutathione, vitamin C, and vitamin E tablets, three times daily, and hydroquinone cream, twice-daily for 12 weeks	NR	0

Xu et al. 2019	Randomized, controlled trial	102 (52E, 50C)	29–53	E: 1–20; C: 1–19	Honghua Xiaoyao tablets, three times daily + tranexamic acid, twice-daily, and hydroquinone cream, twice-daily 12 weeks	Tranexamic acid, twice-daily, and hydroquinone cream, twice-daily 12 weeks	E: 2 × gastrointestinal reaction and 1 × erythema; C: 1 × gastrointestinal reaction, 2 × menoxenia, and 1 × facial tingling	0

Zhu et al. 2016	Randomized, controlled trial	85 (43E, 42C)	20–50 E: 34.67 ± 4.28; C: 34.81 ± 1.15	E: 4.2 ± 1.9; C: 5.3 ± 1.8	Danggui Shaoyao decoction, twice-daily + glutathione tablet, once-daily for 12 weeks	Glutathione tablet, once-daily for 12 weeks	E: 2 × gastrointestinal reaction and 1 × pricking; C: 2 × pricking and 1 × peeling	0

Wang et al. 2019	Randomized, controlled trial	84 (42E, 42C)	23–47 E: 31.54 ± 3.11; C: 30.86 ± 3.25	E: 4.61 ± 1.19; C: 4.58 ± 1.23	Jingtian Quban capsule, twice-daily + glutathione tablet, three times daily for 12 weeks	Glutathione tablet, three times daily for 12 weeks	NR	0

NR: not reported.

**Table 2:** Outcome measures and description of efficacy across studies.

Study	Outcome measures (measurement points)	Efficacy (PT + RT vs RT)
Chen et al. 2016	Melasma area and color score, physician assessment, and serum levels of *α*-MSH and E2 (12th week)	% of patients with “cure” or “improvement”: clinical response: 85.4% vs. 39.6% (*p* < 0.05); % improvement in melasma area score: 71.7% vs. 33.6% (*p* < 0.05); % improvement in melasma color score: 81.9% vs. 36.2% (*p* < 0.05); % improvement in serum levels of *α*-MSH: 38.2% vs. 2.4% (*p* < 0.05) and E2: 31.6% vs. 19.4% (*p* < 0.05)

Ji et al. 2013	Physician assessment (12th week)	% of patients with “cure” or “improvement”: clinical response: 84% vs. 58% (*p* < 0.05)

Jiang et al. 2019	Physician assessment, recurrence rate (8th and 12th weeks, 3-month follow-up)	At 8th week, % of patients with “cure” or “improvement”: clinical response: 42.2% vs. 37.8% (*p* > 0.05). At 12th week, % of patients with “cure” or “improvement”: clinical response: 66.7% vs. 55.6% (*p* < 0.05), recurrence rate: 8.9% vs. 20% (*p*=0.025)

Lu et al. 2017	Physician assessment (12th week)	% of patients with “cure” or “improvement”: clinical response: 85.4% vs. 61.0% (*p* < 0.05)

Luo et al. 2019	Physician assessment, MASI score, and serum levels of sex hormones (12th week)	% of patients with ‘‘cure” or ‘‘improvement”: clinical response: 60.0% vs. 26.9% (*p* < 0.05); % improvement in MASI score: 77.1% vs. 41.2% (*p* < 0.05); % improvement in serum levels of E2: 71.6% vs. 48.4% (*p* < 0.05), testosterone: 133.8% vs. 52.3% (*p* < 0.05), progesterone: 1.4% vs. 1.4% (*p* > 0.05), FSH: 62.0% vs. 36.7% (*p* < 0.05), and LH: 53.0% vs. 30.8% (*p* < 0.05)

Wang et al. 2018	Physician assessment, MASI score, DLQI, serum levels of SOD, and MDA (12th week and 3-month follow-up)	At 12th week % of patients with “cure” or “improvement”: clinical response: 50.0% vs. 30.0% (*p* < 0.05); % improvement in MASI score: 76.3% vs. 48.5% (*p* < 0.05); % improvement in DLQI: 67.6% vs. 51.4% (*p* < 0.05); % improvement in serum levels of SOD: 86.3% vs. 48.8% (*p* < 0.05) and MDA: 55.2% vs. 36.5% (*p* < 0.05); 3-month follow-up % improvement in MASI score: 76.1% vs. 42.1% (*p* < 0.05); % improvement in DLQI: 66.1% vs. 24.0% (*p* < 0.05)

Wang et al. 2019	Physician assessment and serum levels of E2, LH, and FSH (12th week)	% of patients with “cure” or “improvement”: clinical response: 81.0% vs. 52.4% (*p* < 0.05); improvement in serum levels of E2, LH and FSH (*p* < 0.05)

Xie et al. 2016	Physician assessment, skin lesion score, DLQI, and serum levels of E2, progestogen, SOD, MDA, and LPO (12th week)	% of patients with “cure” or “improvement”: clinical response: 68.3% vs. 52.4% (*p* < 0.05); % improvement in skin lesion score: 75.5% vs. 64.3% (*p* < 0.01); % improvement in DLQI: 79.5% vs. 64.4% (*p* < 0.01); % improvement in serum levels of E2: 19.1% vs. 8.0% (*p* < 0.01), progestogen: 27.9% vs. 13.0% (*p* < 0.01), SOD: 36.7% vs. 16.1% (*p* < 0.01), MDA: 26.3% vs. 21.5% (*p* < 0.01), and LPO: 41.9% vs. 25.9% (*p* < 0.01)

Xu et al. 2019	Physician assessment and skin lesion score (12th week)	% of patients with “cure” or “improvement”: clinical response: 80.8% vs. 62.0% (*p* < 0.05); % improvement in skin lesion score: 55.1% vs. 46.4% (*p* < 0.05)

Zhu et al. 2016	Physician assessment, skin lesion score, and serum levels of SOD and MDA (12th week)	% of patients with “cure” or “improvement”: clinical response: 67.4% vs. 50.0% (*p* < 0.05); % improvement in skin lesion score: 45.4% vs. 31.9% (*p*=0.045); % improvement in serum levels of SOD: 34.0% vs. 7.0% (*p* < 0.001) and MDA: 41.1% vs. 27.5% (*p*=0.001)

PT + RT: phytotherapy plus routine therapy, RT: routine therapy, E2: estradiol, MSH: melanocyte-stimulating hormone, FSH: follicle-stimulating hormone, LH: luteinizing hormone, DLQI: dermatology life quality index, and LPO: lipid hydroperoxide.

## Data Availability

The data are available on request to the corresponding author.

## Supplementary Materials

Supplementary Materials Figure S1: funnel plot of trials reporting response rate outcome. Figure S2: forest plot of Melasma Area and Severity Index (MASI). Figure S3: funnel plot of trials reporting adverse events (AEs).
