# Supplementary material for: Efficacy and Safety of Oral Herbal Drugs Used as Adjunctive Therapy for Melasma: A Systematic Review and Meta-Analysis of Randomised Controlled Trials
Source: Evid Based Complement Alternat Med. 2021 Dec 6;2021:9628319. doi: 10.1155/2021/9628319 (PMC8668325; doi:10.1155/2021/9628319)
Supplement: Supplementary Materials — Figure S1: funnel plot of trials reporting response rate outcome. Figure S2: forest plot of Melasma Area and Severity Index (MASI). Figure S3: funnel plot of trials reporting adverse events (AEs). [file 9628319.f1.pdf]

Supplementary Materials

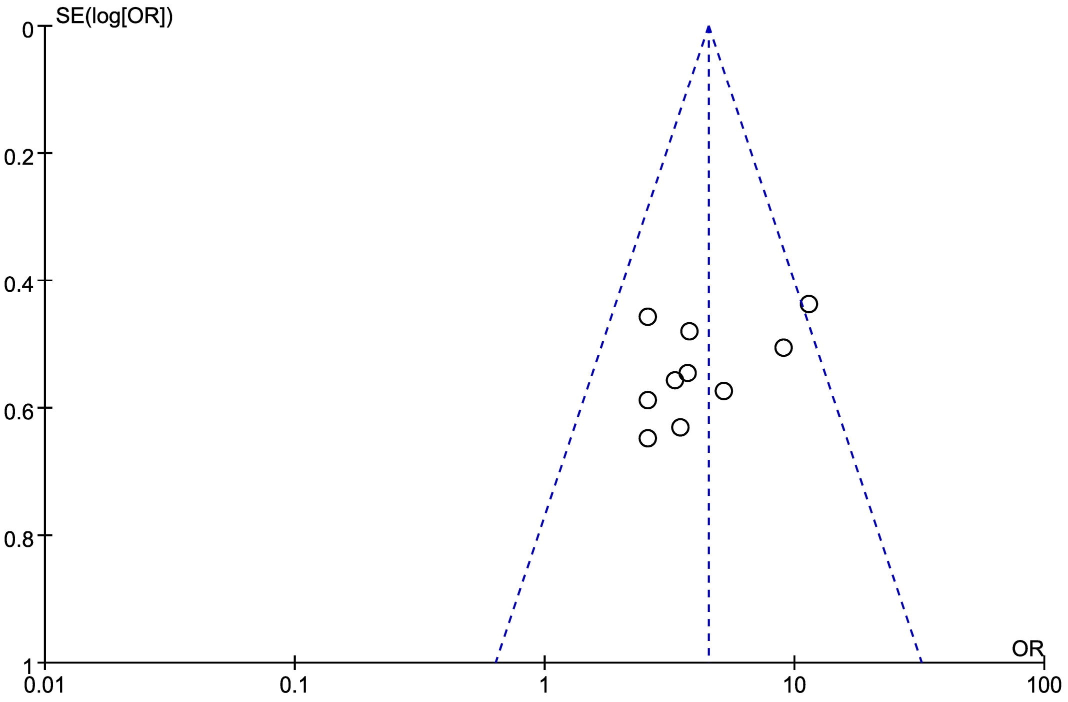

Figure S1 Funnel plot of trials reporting response rate outcome.

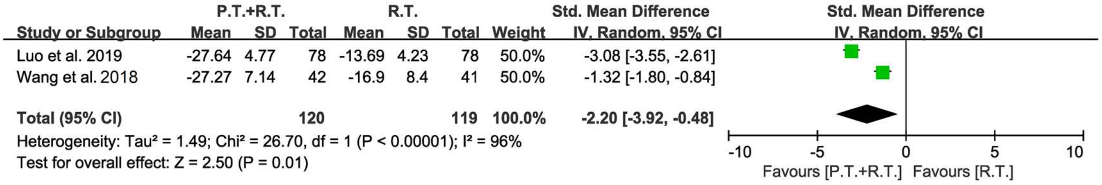

Figure S2 Forest plot of Melasma Area and Severity Index (MASI) in overall analysis. P.T.+R.T.=phytotherapy plus routine therapy; R.T.=routine therapy.

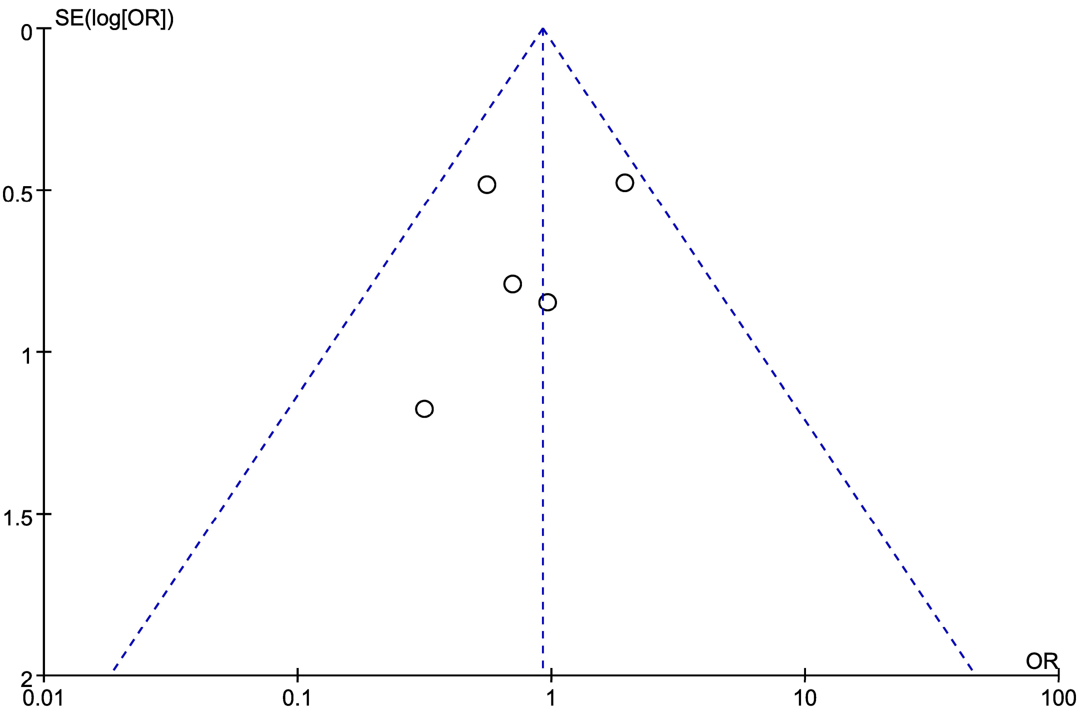

Figure S3 Funnel plot of trials reporting adverse events (AEs).
